# Influence of the physicochemical characteristics of mosquito breeding sites in domestic environments on the distributions of *Anopheles*, *Aedes* and *Culex* mosquitoes in Benin

**DOI:** 10.1186/s41182-025-00786-6

**Published:** 2025-07-30

**Authors:** Isidore Hoyochi, Germain Gil Padonou, Tatchémè Filémon Tokponnon, Alphonse Keller Konkon, David Mahouton Zoungbédji, Albert Sourou Salako, Brice Dangnon, A. Virgile Onésime Akowanou, Luc Olivier Sintondji, Edmond Sossoukpe, Lamine Baba-Moussa, Martin Codjo Akogbéto

**Affiliations:** 1 Centre de Recherche Entomologique de Cotonou (CREC), 06 BP 2604, Cotonou, Benin; 2 Faculté des Sciences et Techniques, Université d’Abomey-Calavi, Abomey-Calavi, Benin; 3 Institut Supérieur des Sciences et de Médecine Vétérinaire (ISSMV) de Dalaba, Dalaba, Guinea; 4 Laboratoire des Sciences et Techniques de l’Eau et de l’Environnement (LSTEE), Institut National de L’eau, University d’Abomey-Calavi (UAC), 01 BP 1446, Abomey-Calavi, Benin; 5 Laboratoire de Biologie et de Typage Moléculaire en Microbiologie, Département de Biochimie et de Biologie Cellulaire, Université d’Abomey-Calavi, Abomey-Calavi, Benin; 6 Laboratoire de Recherches sur les Zones Humides (LRZH), Département de Zoologie, Faculté des Sciences et Techniques, Université d’Abomey-Calavi, Abomey-Calavi, Benin; 7 Laboratoire d’hydrologie Appliquée, Institut National de l’Eau, Centre d’Excellence Africain pour l’Eau et l’Assainissement, Université d’Abomey-Calavi, Abomey-Calavi, Benin; 8 Ecole Polytechnique d’Abomey Calavi, Université d’Abomey-Calavi, Abomey-Calavi, Benin

**Keywords:** Breeding sites, Physicochemical characteristics, Domestic environments, Culicidae, Bénin

## Abstract

**Background:**

Malaria, dengue and lymphatic filariasis are diseases transmitted by *Anopheles*, *Aedes* and *Culex* mosquitoes, respectively. These mosquitoes have evolved and adapted to environmental conditions and human lifestyles, providing them with a variety of breeding sites. This study aimed to determine the influence of the physicochemical characteristics of breeding sites on the distribution of *Anopheles*, *Aedes* and *Culex* mosquitoes in Benin.

**Methods:**

The collections took place from January to November 2025 and lasted 9 months. Mosquito larvae were collected from 11 municipalities in Benin. Physicochemical parameters such as pH, temperature, salinity, total dissolved solids, conductivity, dissolved oxygen and turbidity were measured in situ during larval sampling. Variations among the physicochemical parameters were assessed via Chi-square multiple comparisons of proportions and the least significant difference (LSD) test following analysis of variance (ANOVA). Correlations between physicochemical variables were analyzed via principal component analysis (PCA).

**Results:**

The results revealed that domestic containers and tires were the most common indoor and outdoor breeding sites for human dwellings, respectively. The pH levels slightly varied across the different breeding sites but generally remained nearly neutral. The temperatures were relatively consistent among the habitats, averaging approximately 30 °C. Salinity levels were close to zero at most *Aedes* and *Anopheles* breeding sites, whereas *Culex* larvae were commonly associated with sites with salinity values close to one. The total dissolved solids and conductivity varied considerably among the sites. Dissolved oxygen was positively correlated with the presence of *Anopheles* larvae. Both *Aedes* and *Anopheles* are typically found in habitats with low turbidity, whereas *Culex* larvae are associated with highly turbid environments, with turbidity ranging from 10 to 858 NTU. Principal component analysis revealed strong positive correlations between conductivity, salinity, and TDS; weak correlations between turbidity and temperature; and negative correlations between dissolved oxygen, conductivity, and salinity.

**Conclusion:**

This study highlights the importance of the physicochemical properties of breeding sites in the distribution of primary vector mosquito species. The prevalence of domestic containers and tires as breeding sites underscores the necessity of targeted interventions in these habitats, both indoors and outdoors of human dwellings.

**Supplementary Information:**

The online version contains supplementary material available at 10.1186/s41182-025-00786-6.

## Background

Water is an essential resource for life. But inadequate management can turn it into an environmental factor that promotes the proliferation of insects, particularly mosquitoes that transmit vector-borne diseases [1]. Mosquitoes are holometabolous organisms with a life cycle that includes two distinct phases: an aquatic larval stage and a terrestrial (aerial) adult stage [2]. The aquatic larval phase begins with eggs that hatch into larvae, which then develop into pupae before emerging as adults during the terrestrial (aerial) phase [2]. In their adult stage, mosquitoes serve as primary vectors of several human diseases [3], including malaria, dengue, and lymphatic filariasis. These vector-borne diseases continue to pose significant global health challenges. For example, it is estimated that there will be 263 million cases of malaria are expected worldwide in 2023 [4]. The WHO African Region bears the greatest burden of the disease, accounting for 94% of malaria cases and 95% of malaria deaths [4]. Benin is one of the endemic countries that has recorded over 2,656,855 malaria cases and 1914 associated deaths [5]. Dengue is a potentially fatal viral disease that can overwhelm health systems and disrupt national economies [6, 7]. It is endemic in tropical and subtropical regions around the world [7]. In Africa, 171,991 cases of dengue fever and 753 dengue-related deaths have been reported in countries [8], including Burkina Faso, Cape Verde, Senegal, and Nigeria [9–12]. In Benin, around 30 suspected cases of dengue fever were reported in 2019 [13]. Furthermore, studies conducted in Benin have shown the presence of *Aedes albopictus* and *Ae. aegypti* in several southern Beninese localities [14–16]. In addition, mosquitoes of the Culex genus transmit serious diseases, such as Japanese encephalitis and lymphatic filariasis [17, 18]. More than 120 million people in 81 countries worldwide are infected with Lymphatic filariasis [17]. Additionally, there are 100,000 clinical cases and 30% of deaths occur each year worldwide due to Japanese encephalitis [19]. Vector control remains a key strategy in the fight against vector-borne diseases [20–22]. The most widely implemented approaches, include long-lasting insecticide-treated nets (LLINs) and indoor residual spraying (IRS). These approaches target the adult stage of the mosquito [23–25]. Insecticide-treated nets (ITNs) remain one of the most effective interventions for malaria control [26–28], and their use has increased significantly in sub-Saharan Africa over the past decades [29]. Despite these control efforts, the number of cases and deaths from mosquito-borne diseases continues to rise, as vector species increasingly adapt to available control tools. This adaptation presents a significant challenge to the effective control of vector-borne diseases [30]. Therefore, implementing alternative control strategies that target mosquito larvae is essential. Larval control is advantageous because larvae are typically confined to specific, stable breeding sites that occupy relatively small and identifiable areas, unlike adult mosquitoes, which are dispersed over wide and difficult-to-reach locations [31]. Moreover, the abundance and distribution of malaria and dengue vectors are closely linked to the characteristics of the breeding sites that support their development [2]. Several physicochemical factors in water bodies influence both female oviposition site selection and the survival and development of mosquito larvae [2, 32]. These factors influence key physiological and morphological traits of adult mosquitoes, including body size, fecundity, longevity, and vectorial capacity [2]. Furthermore, a better understanding of the bioecological characteristics of mosquito vectors, along with the physicochemical properties of their breeding sites, is essential for effective vector control [3, 33]. Physicochemical parameters such as temperature, dissolved oxygen, pH, conductivity, salinity, total dissolved solids (TDS), and turbidity provide valuable insights into the ecological dynamics and distribution patterns of mosquito species [34, 35]. Despite their importance, few studies in Benin have investigated the role of these environmental factors in mosquito breeding. Therefore, this study aims to assess the influence of the physicochemical characteristics of breeding sites on the distributions of *Anopheles*, *Aedes*, and *Culex* mosquito species in Benin.

## Methods

### Study area

**Fig. 1:**
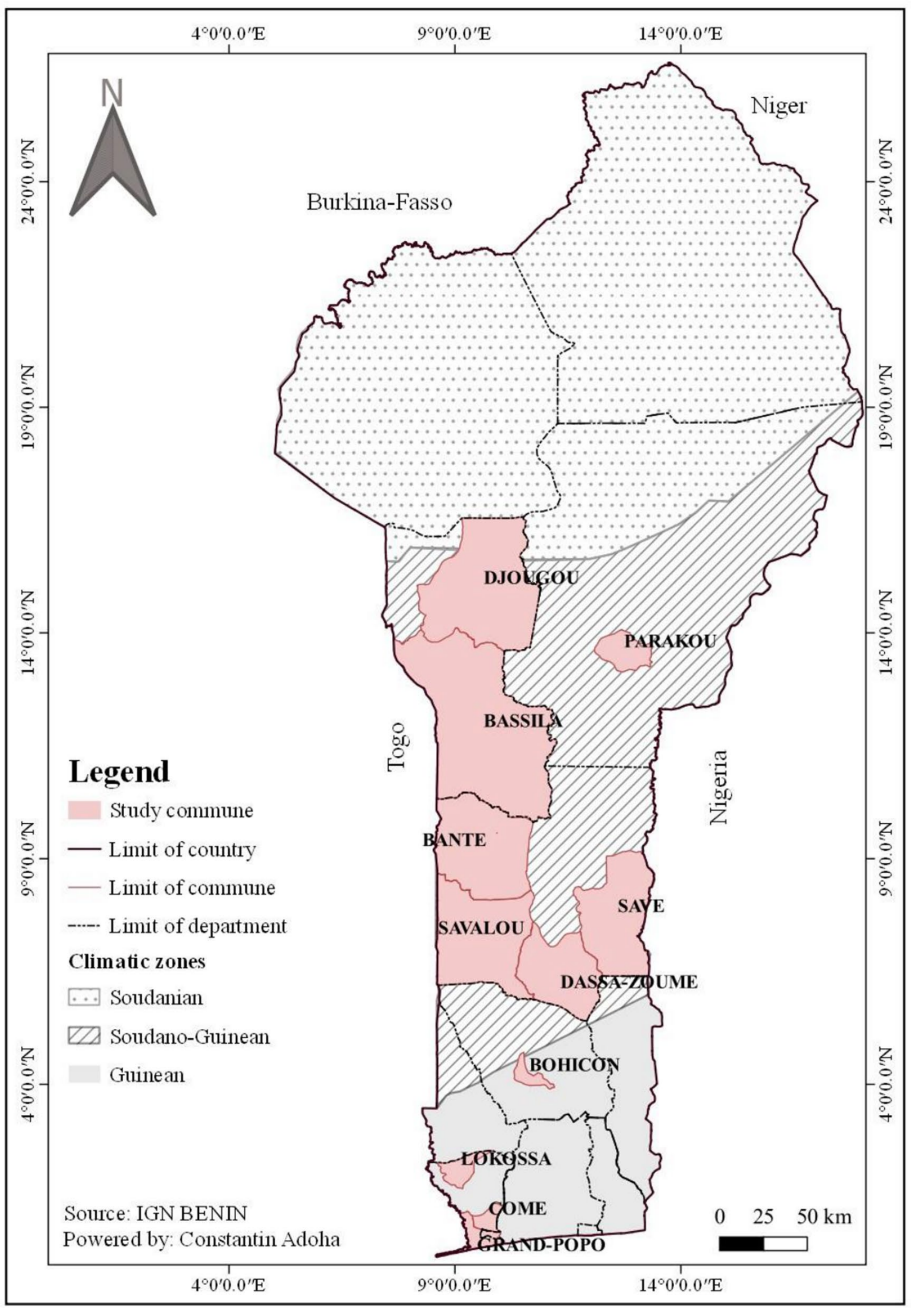
Map of Benin showing the study areas

This research was carried out in 11 municipalities in Benin (Fig. 1). These include the municipalities of Comé, Grand-Popo, Lokossa, Bohicon, Dassa-Zoumé, Savalou, Bantè, Bassila, Djougou, Savè and Parakou. The municipalities of Comé, Grand-Popo, and Lokossa are located in southern Benin. Central Benin is represented by Bohicon, Dassa-Zoumé, Savalou, and Bantè, whereas Bassila, Djougou, Savè, and Parakou represent the northern part of the country. The municipalities of Comé, Grand-Popo, Lokossa, and Bohicon are located in the Guinean climate zone. This region has a subequatorial climate, featuring two rainy and two dry seasons. These conditions favor dense vegetation and diverse agriculture. These cities’ economies are based on fishing, agriculture, and trade. According to the 2013 General Population and Housing Census (RGPH-4), Comé had a population of 79,989, Lokossa had 104,428, Bohicon had 171,781, and Grand-Popo had 76,597. The municipalities of Dassa-Zoumé, Savalou, Bantè, Bassila, Djougou, and Parakou are in the Sudano-Guinean climate zone. This zone is marked by a rainy season and a dry season. It is characterized by forest and wooded savanna vegetation, trees, and shrubs that grow in clayey-sandy, lateritic soils. According to the 2013 census (RGPH-4), Dassa-Zoumé had 112,122 inhabitants; Savalou, 144,549; Bantè, 107,181; Bassila, 130,091; Djougou, 267,812; Parakou, 255,478; and Savè, 87,177. The populations of these areas are accustomed to storing rainwater in cisterns and jars, as well as storing well water in cans, buckets, and barrels for domestic use.

### Mosquito sampling techniques

#### We contacted and obtained consent from each individual in each household

Before collecting larvae from breeding sites in and around homes, we obtained consent from the neighborhood chief, followed by the head of each household. A community liaison guided the Centre de Recherche Entomologique de Cotonou (CREC) team throughout the activity of collecting larvae from households.

#### Larval survey

The collections took place from January to November 2025 and lasted 9 months. Mosquito larvae were collected from 11 municipalities in Benin. In each of the municipalities, a total of 12 households were selected from two neighborhoods, one central and one peripheral. Households were randomly selected within each neighborhood. All domestic and peridomestic water-holding containers (e.g., jars, canaries, water tanks, basins, barrels, tires, and abandoned car bodies) that are likely to harbor mosquito larvae were inspected. During the inspections, larvae and nymphs were collected from containers found to be positive. Each sample was labeled and transported to the Centre de Recherche Entomologique de Cotonou (CREC) insectarium for rearing. These larvae were raised in the CREC insectarium under standard temperature and humidity conditions (26°–30 °C and 60–90% relative humidity). The larvae were fed cat food, which is rich in protein and minerals. Each container was covered with mosquito netting and stored in a room with a relative humidity between 70 and 80% and a temperature between 25 and 30 °C. When the pupae appeared, they were transferred to a 30-cm cube cage to collect the adult mosquitoes as they emerged and prevent crop loss. The adults that emerged from the larvae collected in the field and placed in cages were fed a 10% sucrose solution. After adult emergence, mosquitoes are identified morphologically via the identification keys of Edwards [36], Yiau-Min [37], and Coetzee [38]. The breeding habitats were grouped into five categories: domestic containers (buckets, jars, canaries, barrels, cans, flower pots, drinking troughs); tires; discarded containers (plastic bags, tin cans); natural containers (tree holes, vines); and other sites (abandoned cars, mortars, wheelbarrows, fruit bowls).

#### Measurement of the physicochemical parameters of water from mosquito larval habitats

The physicochemical parameters of water in mosquito larval habitats, including pH, temperature (degrees Celsius), salinity (grams per liter), total dissolved solids (parts per million), conductivity (microsiemens per centimeter), dissolved oxygen (milligrams per liter) and turbidity in nephelometric turbidity units (NTUs), were measured directly in the field (in situ). We measured the pH, temperature, salinity, total dissolved solids (TDS), and conductivity via a multifunction EC/pH meter (model EC5). We measured the dissolved oxygen content via an oxygen meter (model DO9100), and we measured the turbidity via a turbidity meter (model ZD-10A).

### Statistical analysis

The Chi-square multiple comparison of proportions test was used to compare the distributions of the different types of sites found across the surveyed locations. If the *p* value <0.05, then there is a significant difference between the elements being compared. Variations in physicochemical parameters were assessed via analysis of variance (ANOVA) followed by the least significant difference (*p* < 0.05) post hoc test. Principal component analysis (PCA) was employed to evaluate correlations between physicochemical variables and larval density. All the statistical analyses were conducted via R software (version 4.0.3), and graphs were generated with GraphPad Prism 8.

## Results

### Typology of Culicidae breeding sites

**Fig. 2:**
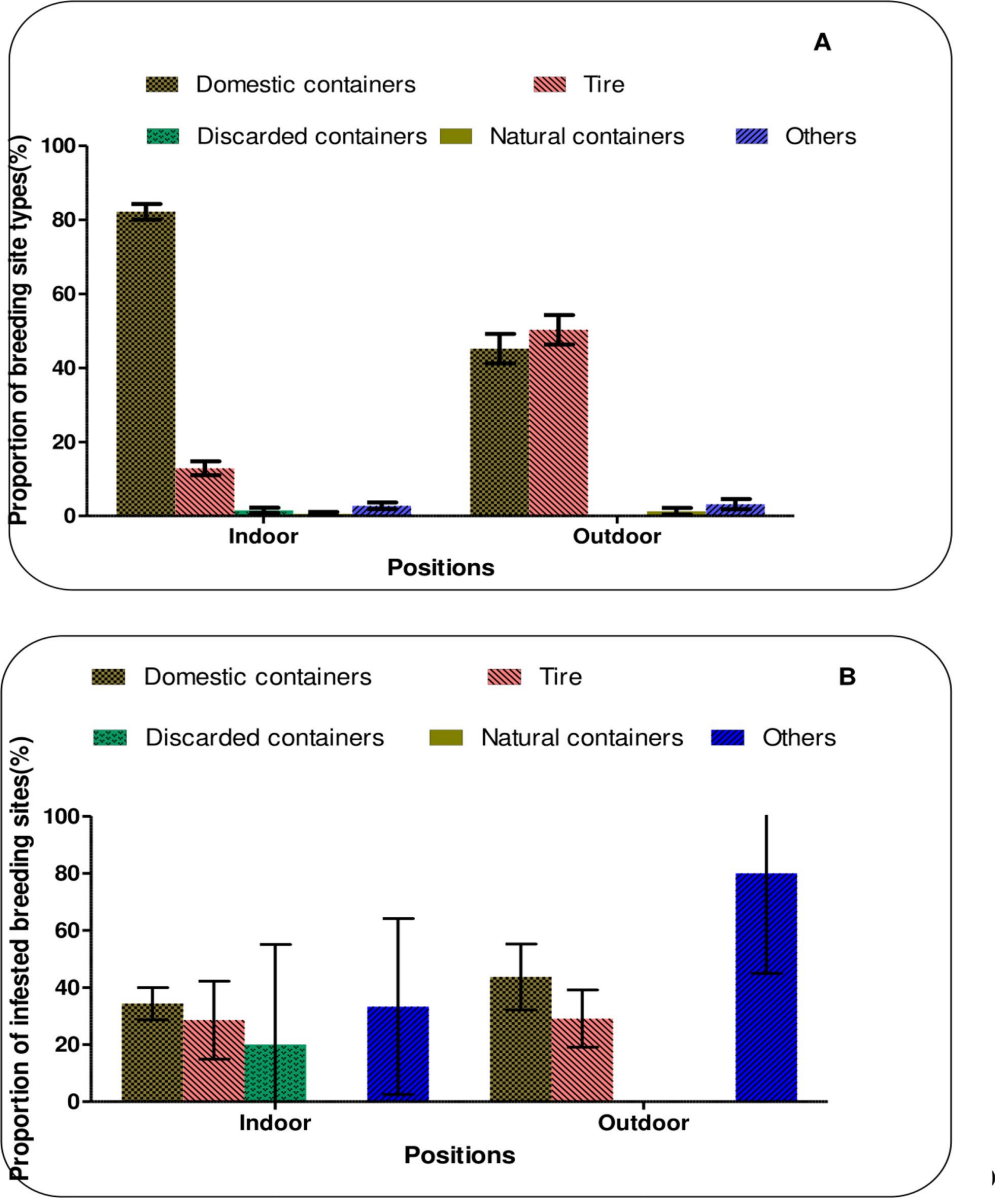
Typology of the breeding sites (**A**) and infested sites (**B**) both indoors and outdoors

Figure 2 shows the typology of Culicidae breeding sites found both indoors and outdoors. A total of 326 potential breeding sites were inspected indoors, of which 108 (33.1%) were positive for mosquito larvae. Outdoors, 157 sites were sampled, with 58 (36.9%) found to be infested with *Aedes* spp., *Anopheles* spp. and *Culex* spp. larvae. Domestic containers were the most common and heavily infested category of breeding sites in both settings, followed by tires, which also harbored significant numbers of mosquito larvae. Overall, Culicinae mosquitoes of the genera *Aedes* and *Culex* were more abundant, accounting for 98% of the larvae collected. Anophelinae were almost absent, accounting for only 1.05% of the mosquitoes in the breeding sites (Table 1).

**Table 1:** Abundance of Culicidae by type of breeding site

Breeding habitats	*Anophelinae*	*Culicinae*		Total
	*%(n/N)_Anopheles* spp.	*%(n/N)_Aedes* spp.	*%(n/N)_Culex* spp.	
Water trough	–	100 (5/5)	–	5
Can	0.90 (12/1319)	84.60 (1116/1319)	14.48 (191/1319)	1319
Kettle	–	85 (51/60)	15 (9/60)	60
Clay pot	–	75 (150/200)	25 (50/200)	200
Water tank	2.24 (18/801)	75.78 (607/801)	21.97 (176/801)	801
tin can	1.59 (5/314)	30.57 (96/314)	67.83 (213/314)	314
Jar	1.88 (64/3400)	70.79 (2407/3400)	27.32 (929/3400)	3400
Mortar	–	29.00 (96/331)	70.99 (235/331)	331
Tire	1.45 (5/3435)	47.45 (1630/3435)	52.40 (1800/3435)	3435
Bucket	1.68 (22/1302)	77.11 (1004/1302)	21.19 (276/1302)	1302
Barrel	0.57 (9/1573)	82.58 (1299/1573)	16.84 (265/1573)	1573
Total	1.05 (135/12,740)	98.94 (12,605/12,740)		12,740

### Determination of the physicochemical characteristics of the water in the breeding areas

**Fig. 3:**
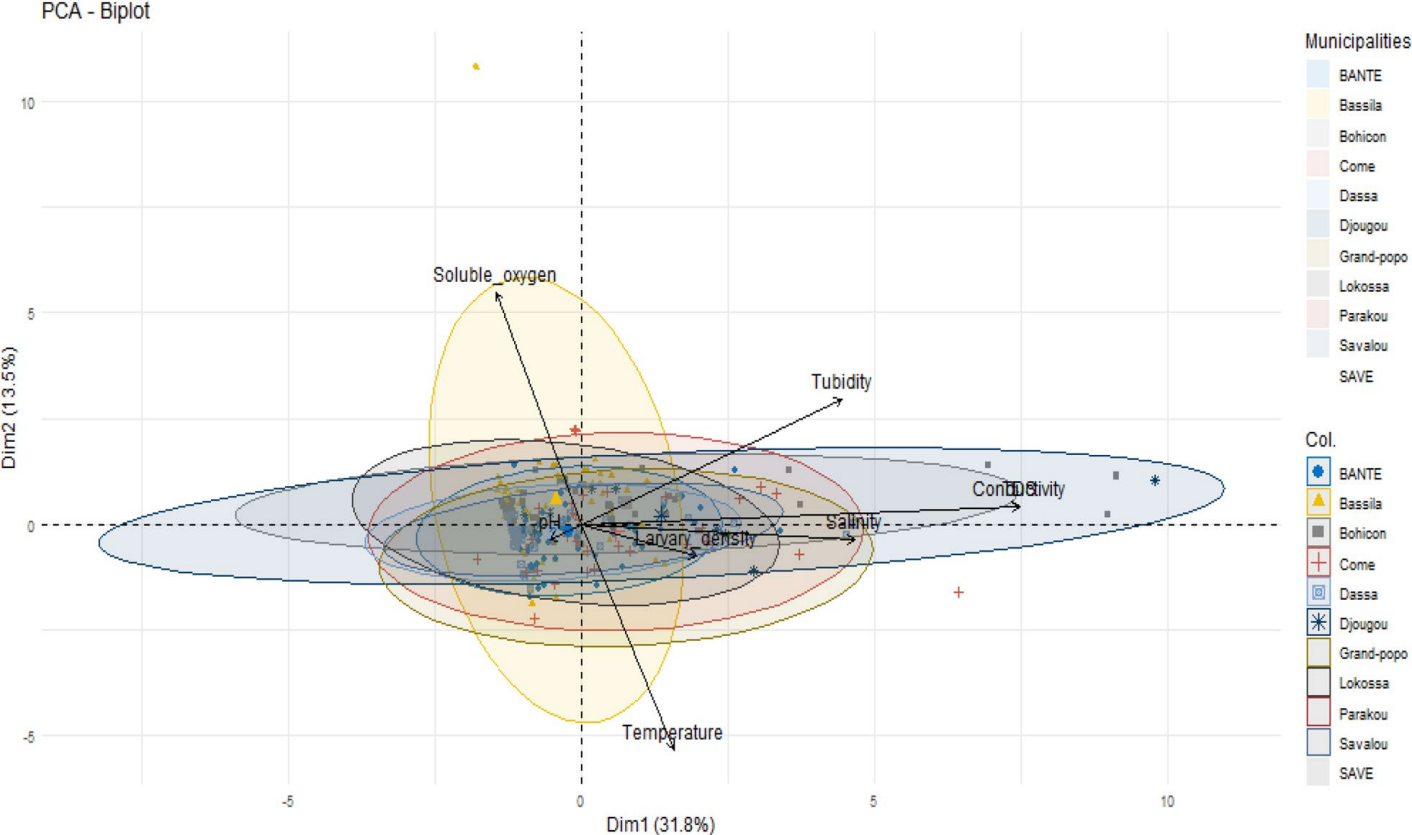
Variation in the physicochemical parameters of the breeding sites by community

Each physicochemical parameter of water has a distinct influence on mosquito larval development. Table 2 presents the values of physicochemical parameters across different breeding sites and mosquito species, with statistically significant differences indicated by superscript letters (a, b, c). In each case, values sharing the same letter are not significantly different, whereas different letters indicate significant variation (*p* < 0.05). The parameters analyzed included pH, temperature, salinity, total dissolved solids (TDS), conductivity, dissolved oxygen (O₂), and turbidity. Overall, the pH values showed minimal variation, remaining close to neutral (approximately 7.0) across most breeding sites and mosquito species. However, an exception was observed in Lokossa, where the pH was markedly higher, averaging 9.5 (Fig. 3).

**Table 2:** Physicochemical properties of the breeding site water by mosquito species and site type

	Number	PH	Temperature	Salinity	TDS	Conductivity	O_2_	Turbidity
Breeding sites								
Water trough	1	8.1^a^	32.3^a^	0.04a^b^	467^ab^	936^ab^	4^abc^	800^a^
*Can*	13	7.58 ± 0.17^ab^	28.43 ± 0.75^a^	0.01 ± 0.01^ab^	167.23 ± 87.95^b^	335.77 ± 177.22^b^	5.08 ± 0.31^a^	129.82 ± 84.05^b^
Clay pot	1	7.21^ab^	33.3^a^	0.03^ab^	388^ab^	774^ab^	1^c^	870^a^
Water tank	11	8.05 ± 0.3^a^	28.42 ± 0.87^a^	0.01 ± 0^b^	102.82 ± 36.26b	205.27 ± 72.33^b^	4.64 ± 0.46^ab^	156.18 ± 96.1^b^
Tin can	4	7.6 ± 0.15^ab^	30.2 ± 0.51^a^	0.05 ± 0.02^a^	562 ± 148.55^a^	1122 ± 298.76^a^	2.4 ± 0.63^c^	640.9 ± 204.17^a^
*Jar*	55	7.63 ± 0.08^ab^	28.95 ± 0.37^a^	0.01 ± 0^b^	133.33 ± 27.71^b^	268.2 ± 55.35^b^	5.02 ± 0.21^a^	77.84 ± 30.68^b^
Mortar	1	8.7^a^	30.8^a^	0.01^ab^	146^b^	293^b^	7.4^a^	820^a^
*Tire*	31	7.77 ± 0.08^ab^	29.45 ± 0.46^a^	0.04 ± 0.01^ab^	307.87 ± 67.9^ab^	617.23 ± 137.25^ab^	3.56 ± 0.24^c^	416.14 ± 71.14^a^
Bucket	21	7.54 ± 0.13^ab^	29.89 ± 0.98^a^	0.01 ± 0^b^	130.52 ± 24.2^b^	260.71 ± 48.19^b^	3.85 ± 0.47^bc^	146.23 ± 64.65^b^
Barrel	13	7.78 ± 0.18^ab^	29.16 ± 0.86^a^	0 ± 0^b^	44.15 ± 11.32^b^	88.38 ± 22.65^b^	5.26 ± 0.24^a^	72.12 ± 60.78^b^
Species								
*Aedes*	79	7.62 ± 0.06^ab^	28.94 ± 0.31^ab^	0.01 ± 0^b^	111.01 ± 15.52^b^	224.72 ± 32.1^b^	4.81 ± 0.16^a^	68.49 ± 24.17^c^
*Aedes_Anopheles*	5	7.46 ± 0.24^ab^	28.74 ± 0.9^ab^	0.02 ± 0.01^b^	209.6 ± 71.62^b^	418.4 ± 142.42^b^	4.66 ± 0.55^ab^	163.6 ± 161.36^bc^
*Aedes_Anopheles_Culex*	2	7.4 ± 0.1^ab^	26.15 ± 0.65^b^	0 ± 0^b^	30 ± 16^b^	60.5 ± 31.5^b^	5.77 ± 0.27^a^	0 ± 0c
*Aedes_Culex*	26	7.96 ± 0.13^a^	29.8 ± 0.71^ab^	0.02 ± 0^b^	220.46 ± 42.13^b^	441.35 ± 84.41^b^	4.34 ± 0.36^ab^	366.43 ± 76.78^ab^
*Anopheles*	3	7.5 ± 0.2^ab^	32.2 ± 1.1^a^	0.01 ± 0.01^b^	87.67 ± 61.28^b^	175.33 ± 122.03^b^	5.3 ± 0.4^a^	14 ± 9.45c
*Anopheles_Culex*	8	7.66 ± 0.11^ab^	28.31 ± 0.98^ab^	0.01 ± 0^b^	111.62 ± 58.42^b^	224.25 ± 117.56^b^	4.15 ± 0.5^ab^	312.5 ± 146.04^ab^
*Culex*	19	7.65 ± 0.13^ab^	29.48 ± 0.65^ab^	0.06 ± 0.02^a^	398.95 ± 124.68^a^	793.32 ± 250.07^a^	3.54 ± 0.54^ab^	464.24 ± 91.24^a^
*Negative*	9	8 ± 0.38^a^	30.06 ± 1.4^ab^	0.02 ± 0.01^b^	266.67 ± 71.35^ab^	532.11 ± 142.79^ab^	3.44 ± 0.68^ab^	281.21 ± 133.84^ab^

In each column, means with the same letter are not significantly different (LSD test, ANOVA)

*TDS* total dissolved solids, *O*_*2*_ dissolved oxygen

The temperatures were relatively consistent across all the study sites, averaging approximately 30 °C. Salinity levels were generally low, approximately 0.02 g/L for most *Aedes* and *Anopheles* breeding sites. In contrast, *Culex* larvae were found in habitats with slightly higher or more variable salinity levels, averaging approximately 0.06 g/L. The total dissolved solids (TDS) and electrical conductivity showed more substantial variability among the sites. *Aedes* and *Anopheles* larvae were typically associated with lower TDS and conductivity values (approximately 103.22 mg/L), whereas *Culex* larvae were predominantly found in habitats with significantly higher TDS and conductivity values (averaging approximately 700 mg/L). Dissolved oxygen levels were also relatively high at breeding sites containing *Anopheles* larvae, with a mean concentration of 5.3 mg/L. With respect to turbidity, *Aedes* and *Anopheles* larvae were found in clear water (5–10 NTU), whereas *Culex* larvae were commonly associated with highly turbid environments, with turbidity levels reaching 858 NTU. Overall, there was minimal variation in physicochemical parameters across the different geographic regions and climatic zones studied (Fig. 3), except for moderate differences in pH and temperature among the communities.

### Relationships between physicochemical parameters related to mosquito breeding sites and mosquito genera

**Fig. 4:**
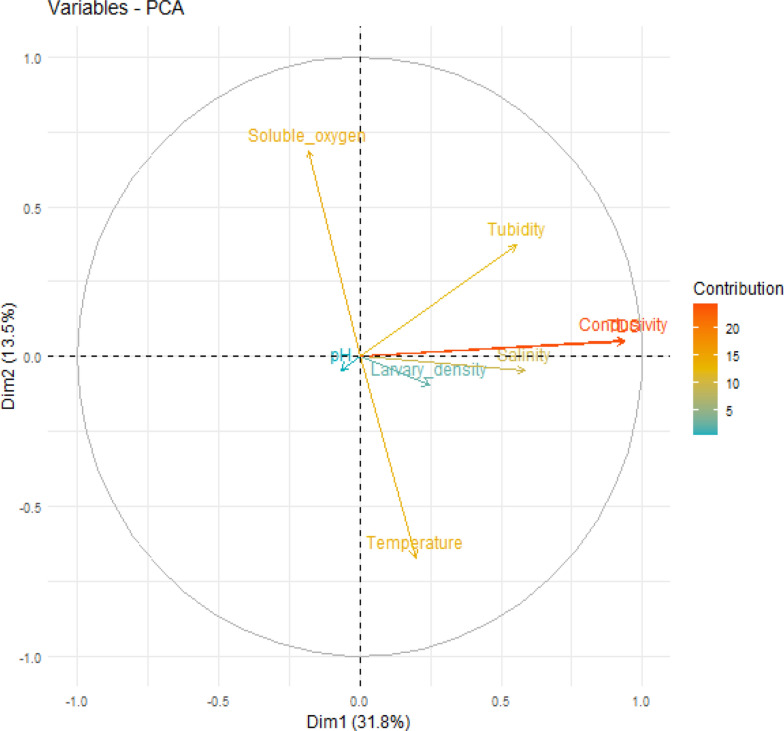
Correlations between physicochemical parameters of mosquito breeding sites [PCA Dim1 × Dim2]

**Fig. 5:**
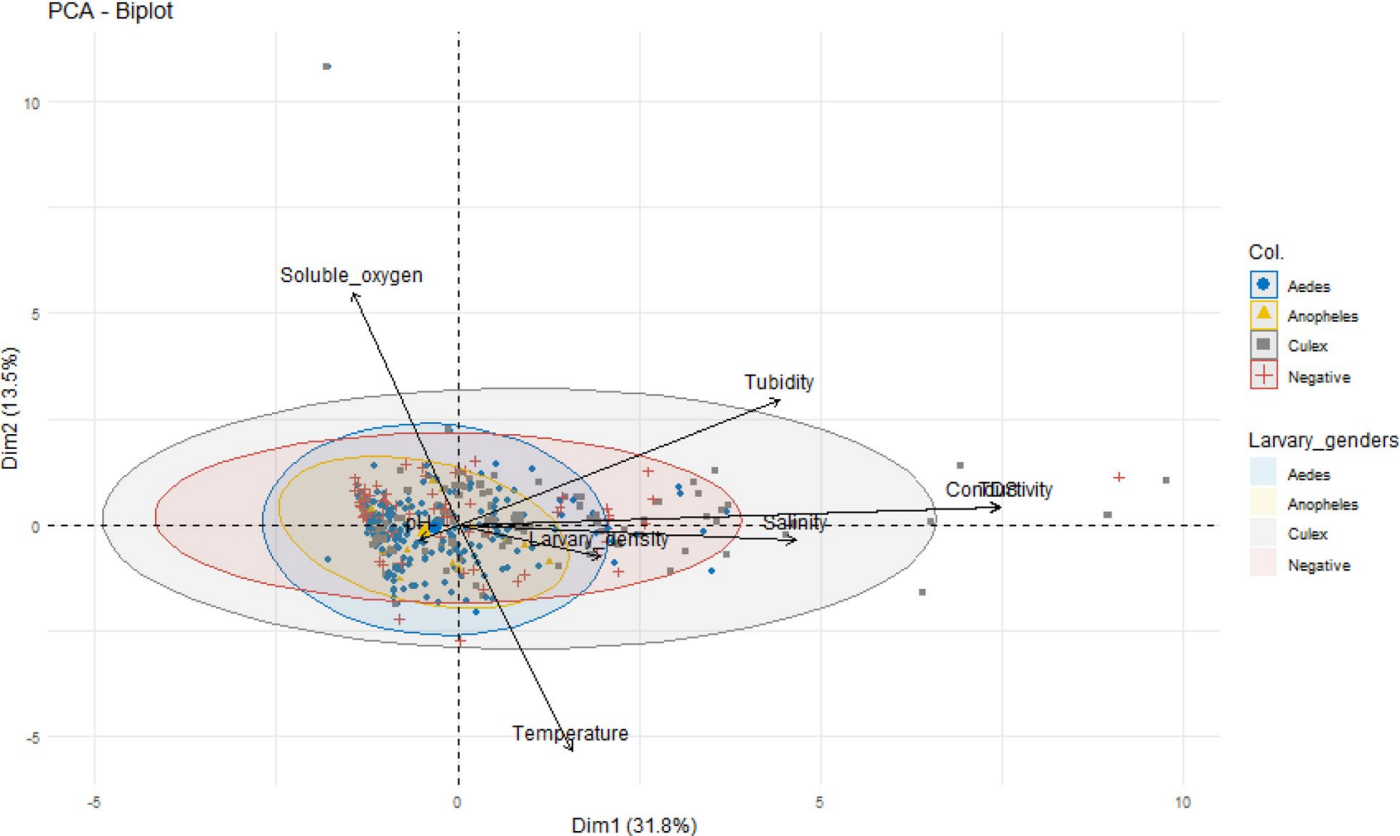
Correlations between physicochemical parameters of the breeding sites and mosquito genera

Principal component analysis (PCA) was conducted to explore the relationships between the physicochemical parameters of the mosquito breeding sites and the presence of different mosquito genera. Eight variables were included in the PCA: pH, temperature, dissolved oxygen (DO), salinity, conductivity, total dissolved solids (TDS) and turbidity (Fig. 4). The first principal component (PC1), which explained 31.8% of the total variance, showed that conductivity, salinity, and TDS were strongly correlated and moved in the same direction. Notably, there was a very strong positive correlation between conductivity and TDS (*r* = 0.99) and a moderate correlation between conductivity and salinity (*r* = 0.40), particularly at breeding sites favorable for *Culex* larval development (Fig. 5). In contrast, *Aedes* and *Anopheles* larvae were associated with breeding habitats characterized by lower levels of conductivity, TDS, and salinity, indicating their preference for less mineralized and less saline environments.

**Fig. 6:**
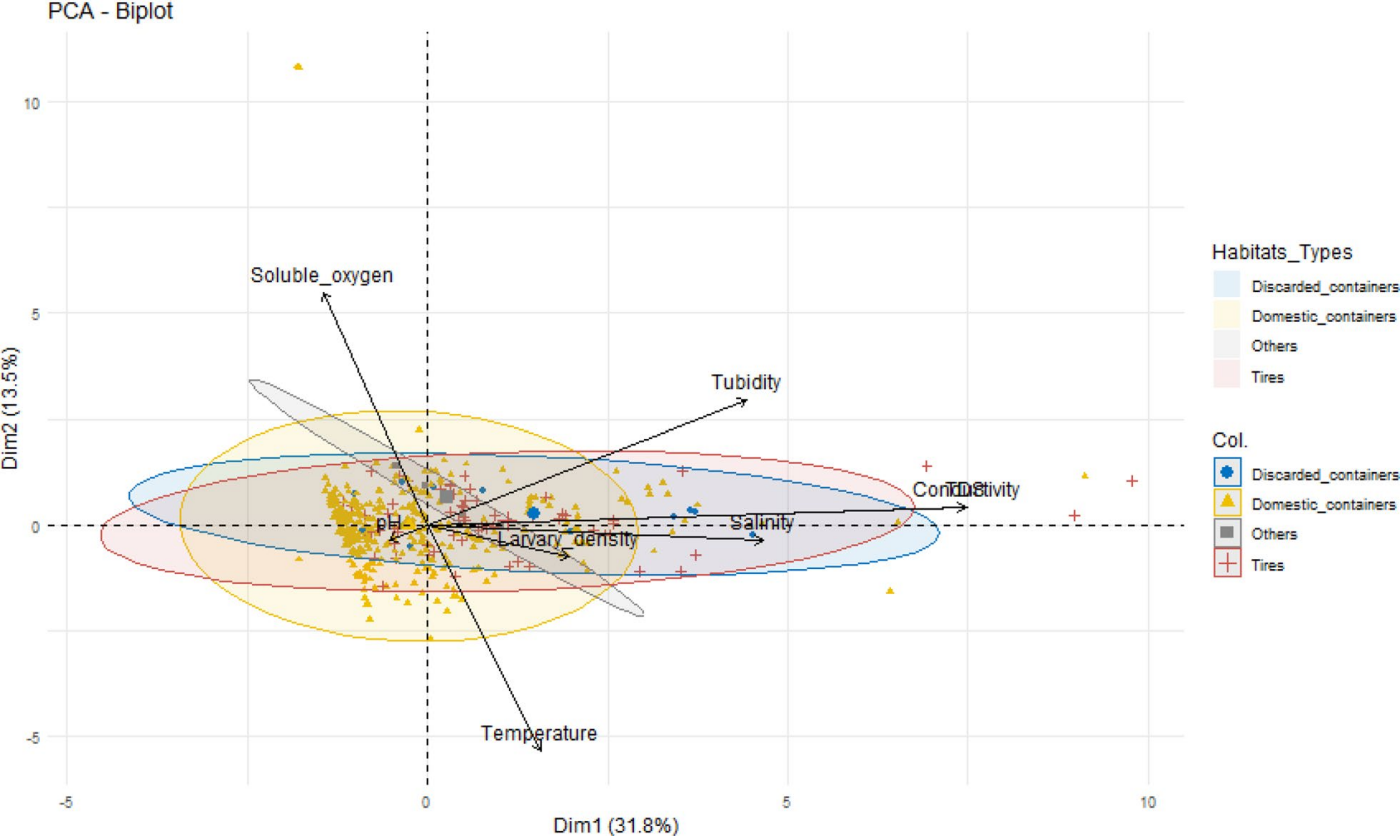
Variation in physicochemical parameters at different types of breeding sites

The analysis revealed weak negative correlations between turbidity and temperature (*r* = −0.006), as well as between pH and both temperature (*r* = −0.01) and turbidity (*r* = −0.05). Dissolved oxygen levels were inversely related to conductivity (*r* = −0.11), salinity (*r* = −0.07), and temperature (*r* = −0.08), suggesting that higher ion content and warmer conditions may reduce oxygen availability in breeding water. Figure 6 shows that conductivity, total dissolved solids (TDS), and salinity were consistently greater in discarded containers and tires. These habitats were more frequently associated with the presence of *Culex* larvae, indicating a preference of this genus for more mineral-rich and turbid environments (Fig. 6).

## Discussion

This study aimed to assess the physicochemical characteristics of mosquito breeding sites and their influence on the distribution of mosquito species across various ecological zones in Benin. Understanding the nature of mosquito breeding sites is fundamental to the design of effective vector control strategies, as it provides insights into the ecological mechanisms that shape mosquito population dynamics and influence vector-borne disease transmission [39]. The findings revealed that domestic containers were the most prevalent and heavily infested larval habitats indoors, whereas tires were the most common and productive sites for mosquito larvae outdoors. These results can be attributed to the widespread practice of water storage due to inadequate access to potable water. Households often store water in buckets, jars, basins, cans, and barrels for domestic purposes, and many of these containers are left uncovered, creating favorable conditions for mosquito oviposition and larval development. Similarly, the abundance of tires outdoors is likely driven by the proliferation of informal mechanics and tire repair shops, which frequently discard used tires in open environments. During the rainy season, these tires collect and retain rainwater, providing ideal microhabitats for mosquitoes, particularly *Aedes* and *Culex* species. These observations are consistent with earlier studies in Benin [14, 16] and São Tomé in Central Africa [40], which also reported high larval densities in domestic containers and tires. Overall, the majority of the breeding sites encountered during this study were artificial in nature and largely originated from human activities. This aligns with findings from other regions, where urbanization, poor waste management and anthropogenic water storage practices have been shown to create and sustain larval habitats [33, 39, 41, 42]. In addition to characterizing the breeding sites, this study identified larvae of *Aedes*, *Anopheles*, and *Culex* mosquitoes, with *Aedes* and *Culex* being the most abundant. *Aedes* larvae are predominantly found in household containers and tires, which are highly suitable habitats for this genus because of their preference for the clean, stagnant water commonly found in domestic environments. This ecological preference accounts for the significantly greater proportion of *Aedes* larvae than *Anopheles* larvae at these sites. These findings are consistent with those of Padonou et al. [14], who reported a predominance of *Aedes* species in similar larval habitats in Benin. Furthermore, the physicochemical parameters of breeding sites significantly influence the survival, development and distribution of mosquito species [2, 32, 43]. Our results revealed minimal variation in pH across different breeding sites, with values generally near neutral. This near-neutral pH is likely due to the predominance of rain, tap, or groundwater at these sites. These findings align with those of previous studies by Iro et al. [32] and Clark et al. [44], who reported that a pH of approximately seven is conducive to egg hatching and larval development. Other investigations have indicated that mosquito larvae survive optimally within a pH range of six and six and eight [45, 46]. The temperature, a critical factor in aquatic insect development [2], was consistent across the breeding sites, ranging from 26 to 32 °C. This range corresponds with the optimal conditions for mosquito larval development reported by Bayoh et al. [47] and is comparable to the ranges observed in studies by Hery et al. [2] (20–36 °C), Hessou-Djossou et al. [33] (24–39 °C) and Iro et al. [32] (19–33 °C). Variations in temperature may be influenced by seasonal timing, time of day, site location, sunlight exposure and differences in measurement instruments [48]. Laboratory studies have confirmed that a stable temperature between 24 and 30 °C promotes the hatching and development of Culicidae larvae [49–51], whereas lower temperatures are generally unfavorable [52]. Despite the known impact of temperature on larval development, similar temperature ranges at both positive and negative breeding sites suggest that temperature may not be a reliable predictor of larval presence [53]. In contrast, salinity appears to influence larval habitat selection [2]. Our findings indicated that *Culex* larvae tolerate higher or lower salinity levels than *Aedes* and *Anopheles*, which prefer breeding sites with salinities below 0.5 g/L, which is consistent with prior research demonstrating their preference for freshwater habitats [2, 33]. However, *Aedes* larvae have been found in waters with salinities of up to 18 g/L in some coastal areas of Brazil and Asia [54], and *Anopheles* larvae show a preference for brackish water in Cotonou [33]. These findings suggest that both species can adapt to unusual breeding sites. The coexistence of these species with *Culex* larvae may be due to *Aedes* and *Anopheles* being osmoconformers, maintaining internal balance in high-salinity environments. Such adaptation could allow their larvae to develop in saline habitats caused by sea level rise, increasing the risk of arbovirus and malaria transmission in coastal zones [2, 54]. *Aedes* and *Anopheles* larvae were found in sediments with low total dissolved solids (TDS) and conductivity, which is consistent with findings of Hessou-Djossou et al. [33] for *Anopheles* in Benin. Other studies on *Aedes* have shown a low variation in conductivity ranging from 50 to 100 μS/cm [2]. Nagamani et al. [55] reported that high conductivity negatively affects *Aedes* larval abundance. Conversely, *Culex* larvae are associated with relatively high conductivity levels, whereas *Anopheles* prefer relatively low conductivity [52, 56]. These patterns improve the understanding of the distribution of Culicidae relative to the physicochemical properties of their breeding sites. With respect to dissolved oxygen, *Aedes* and *Culex* larvae were found across a range of dissolved oxygen levels, whereas *Anopheles* larvae presented slightly higher oxygen concentrations, indicating a preference for well-oxygenated habitats. These findings support previous studies by Lalami et al. [34] and Hessou-Djossou et al. [33], who reported elevated dissolved oxygen in habitats containing *Anopheles* larvae. The density of *Anopheles* larvae is positively correlated with the dissolved oxygen concentration at breeding sites [32, 57, 58]. This difference can be explained by morphological adaptations: *Aedes* and *Culex* larvae possess a siphon that enables atmospheric oxygen intake [59], whereas *Anopheles* larvae lack a well-developed siphon and rely primarily on dissolved oxygen in water for survival. In terms of turbidity, *Aedes* and *Anopheles* larvae were predominantly found at sites with very low turbidity, indicating a preference for clearer, less turbid waters. In contrast, *Culex* larvae preferred highly turbid sites, which often correspond to more polluted water. These observations align with the findings of Iro et al. [32], who reported that *Aedes* larvae thrive in water with turbidity below 5 NTU, and Djègbè et al. [58], who noted that turbid water negatively affects the presence of *Anopheles gambiae*. However, other studies have documented the presence of *Aedes* and *Anopheles* larvae in water with turbidity levels up to 100 NTU [60], suggesting that these species can adapt to suboptimal conditions. Additionally, there was a strong positive correlation between conductivity, salinity, and total dissolved solids (TDS) at sites with *Culex* larvae, which is consistent with the associations of these parameters with water pollution [61]. A weak positive correlation between turbidity and temperature was also observed, suggesting that warmer waters tend to be more turbid, which may further explain the habitat preferences of *Culex* larvae for turbid water compared with the clearer water preferred by *Aedes* and *Anopheles* larvae. There was a moderate negative correlation between pH, turbidity, and temperature, suggesting that higher pH is typically associated with clearer, cooler waters and conditions more favorable for *Aedes* and *Anopheles* larvae. Additionally, conductivity, total dissolved solids (TDS) and salinity were elevated in discarded containers and tires, which are more likely to harbor *Culex* larvae. This pattern may be due to the outdoor location of these sites, which exposes them to higher levels of pollution than domestic containers do.

This study did not evaluate environmental factors such as biotic interactions (e.g., predation or competition), climatic influences, or anthropogenic impacts on Culicidae population dynamics, representing limitations and areas for future research.

## Conclusion

This study identified key mosquito breeding sites and the physicochemical factors influencing larval distribution in domestic settings. Household containers and tires were the most common breeding habitats indoors and outdoors, respectively. *Culex* larvae were associated with saline, turbid waters of high conductivity and TDS, whereas *Aedes* and *Anopheles* larvae were more common in fresher, clearer waters with low conductivity and TDS. *Anopheles* larvae additionally preferred well-oxygenated environments, which often share ecological characteristics with *Aedes*. This ecological overlap may facilitate the coexistence of both species, increasing the potential risk of malaria and dengue coinfections. The findings provide useful insights for the National Malaria Control Programme, the National Programme for the Control of Communicable Diseases, and the National Program for the Elimination of Lymphatic Filariasis to better target vector control interventions for malaria, dengue and lymphatic filariasis.

## Supplementary Information

Additional file 1.

## Data Availability

All the data collected and analyzed in this study and the materials used are available from the corresponding author.

## Abbreviations

PCA: Principal component analysis
CREC: Centre de Recherche Entomologique de Cotonou
WHO: World Health Organization
PH: Hydrogen potential
NTU: Nephelometric turbidity unit
TDS: Total dissolved solids
