# Supplementary figures and images for: Influence of the physicochemical characteristics of mosquito breeding sites in domestic environments on the distributions of Anopheles, Aedes and Culex mosquitoes in Benin
Source: Trop Med Health. 2025 Jul 30;53:100. doi: 10.1186/s41182-025-00786-6 (PMC12308970; doi:10.1186/s41182-025-00786-6)

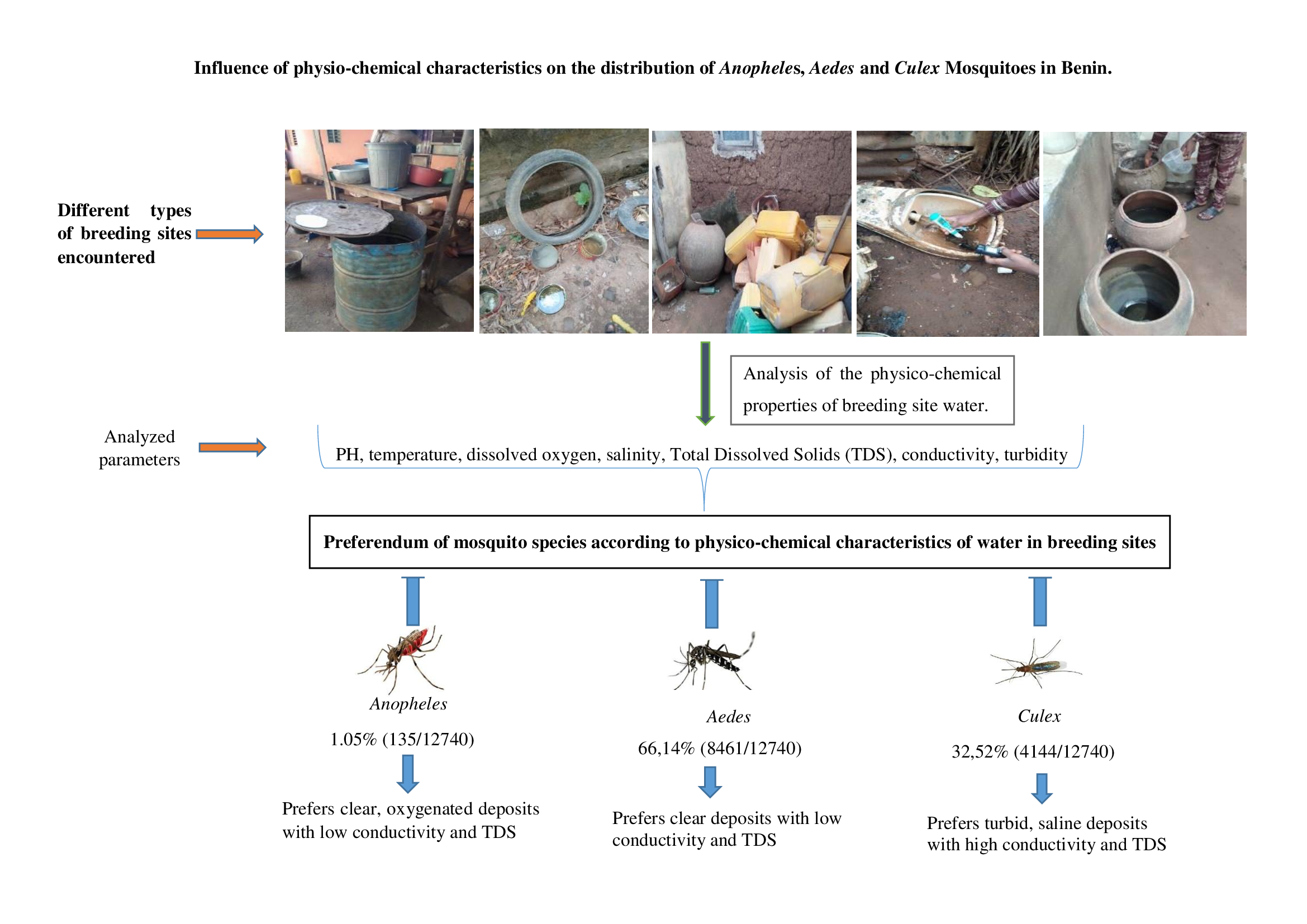

Supplement: Supplementary file 1 — Additional file 1. [file 41182_2025_786_MOESM1_ESM.tiff]
